# Supplementary material for: Amyloid-β fibrils accumulated in preeclamptic placentas suppress cytotrophoblast syncytialization
Source: Life Sci Alliance. 2026 Jan 20;9(4):e202503453. doi: 10.26508/lsa.202503453 (PMC12819053; doi:10.26508/lsa.202503453)
Supplement: Supplementary file 5 [file LSA-2025-03453_SdataF3.2.pptx]

## Slide 1
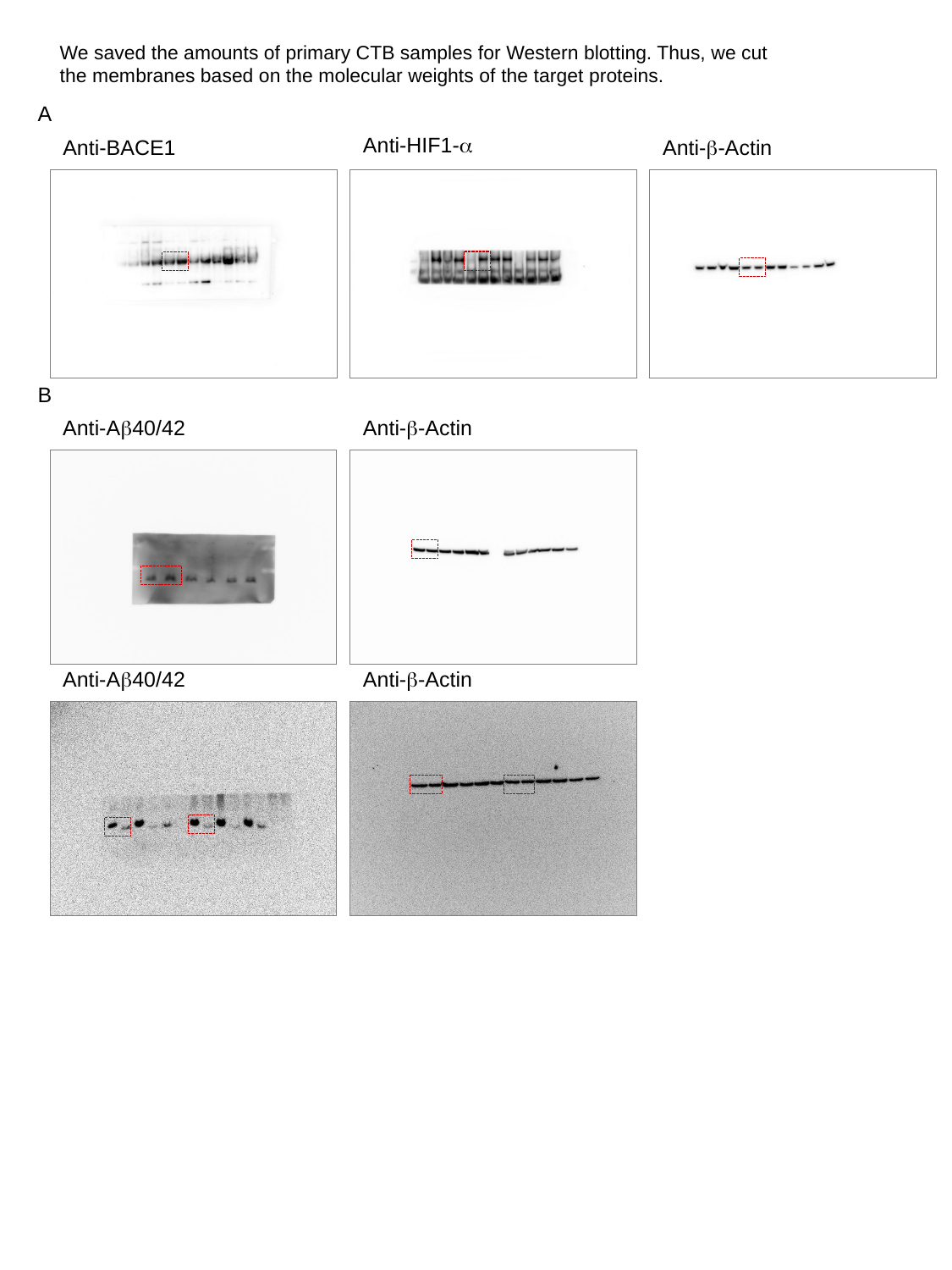

We saved the amounts of primary CTB samples for Western blotting. Thus, we cut the membranes based on the molecular weights of the target proteins.
A
Anti-HIF1-a
Anti-BACE1
Anti-b-Actin
B
Anti-Ab40/42
Anti-b-Actin
Anti-Ab40/42
Anti-b-Actin
