# Supplementary figures and images for: Amyloid-β fibrils accumulated in preeclamptic placentas suppress cytotrophoblast syncytialization
Source: Life Sci Alliance. 2026 Jan 20;9(4):e202503453. doi: 10.26508/lsa.202503453 (PMC12819053; doi:10.26508/lsa.202503453)

## Slide 1
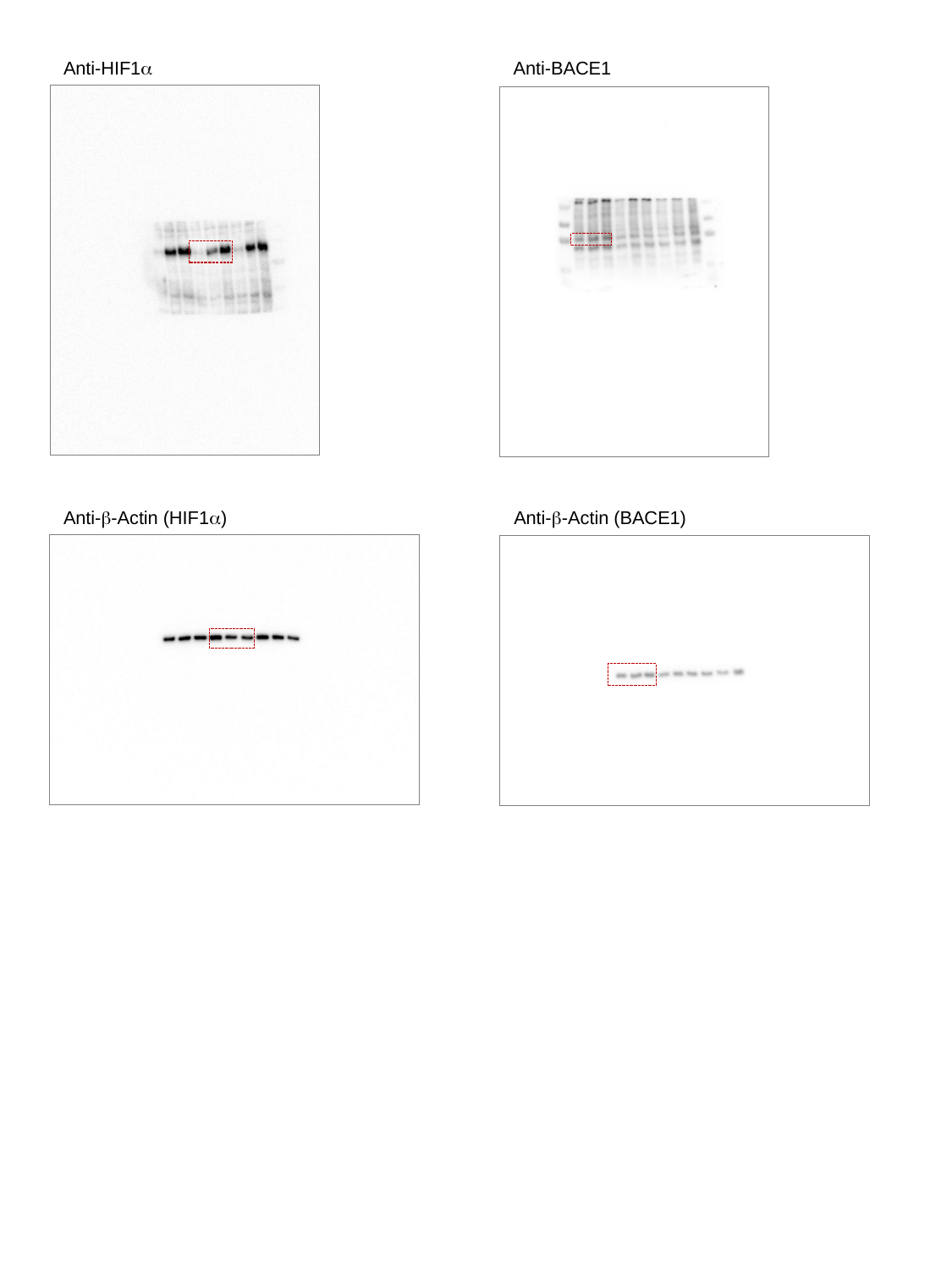

Anti-HIF1a
Anti-BACE1
Anti-b-Actin (HIF1a)
Anti-b-Actin (BACE1)

Supplement: Supplementary file 7 [file LSA-2025-03453_SdataFS2.2.pptx]

## Slide 1
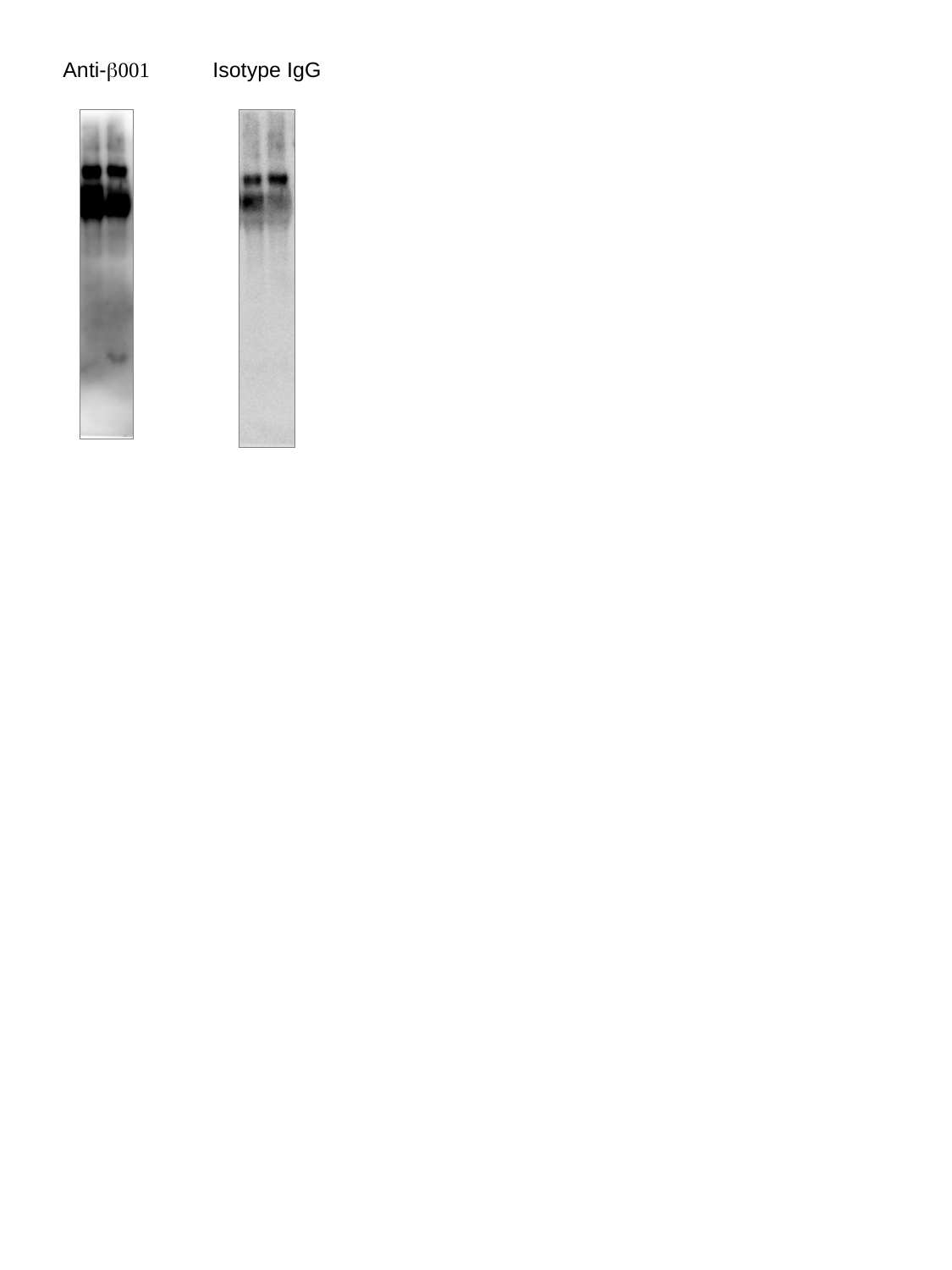

Anti-b001
Isotype IgG

Supplement: Supplementary file 9 [file LSA-2025-03453_SdataFS4.pptx]

## Slide 1
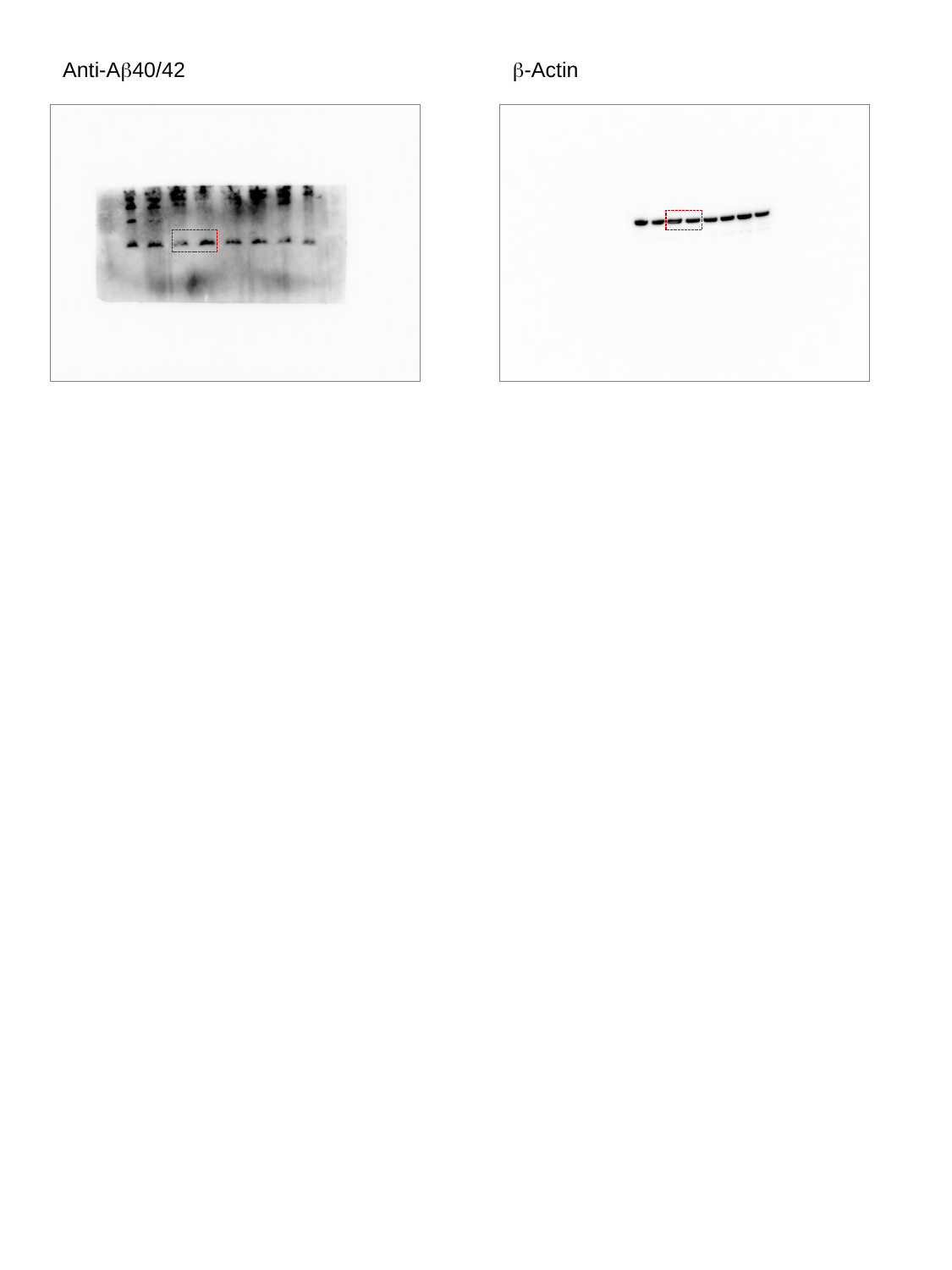

Anti-Ab40/42
b-Actin

Supplement: Supplementary file 11 [file LSA-2025-03453_SdataFS5.2.pptx]

## Slide 1
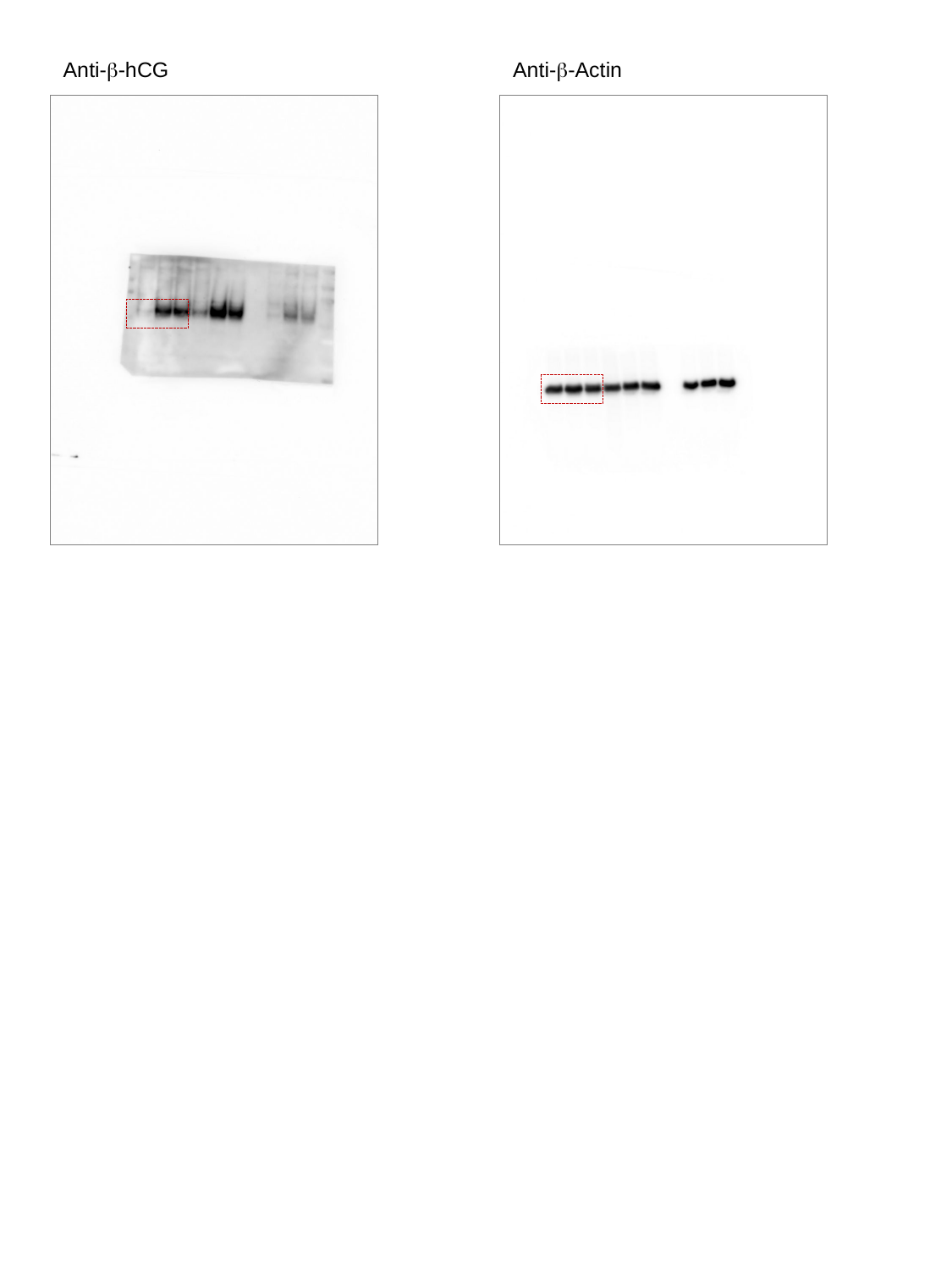

Anti-b-hCG
Anti-b-Actin

Supplement: Supplementary file 15 [file LSA-2025-03453_SdataFS6.2.pptx]

## Slide 1
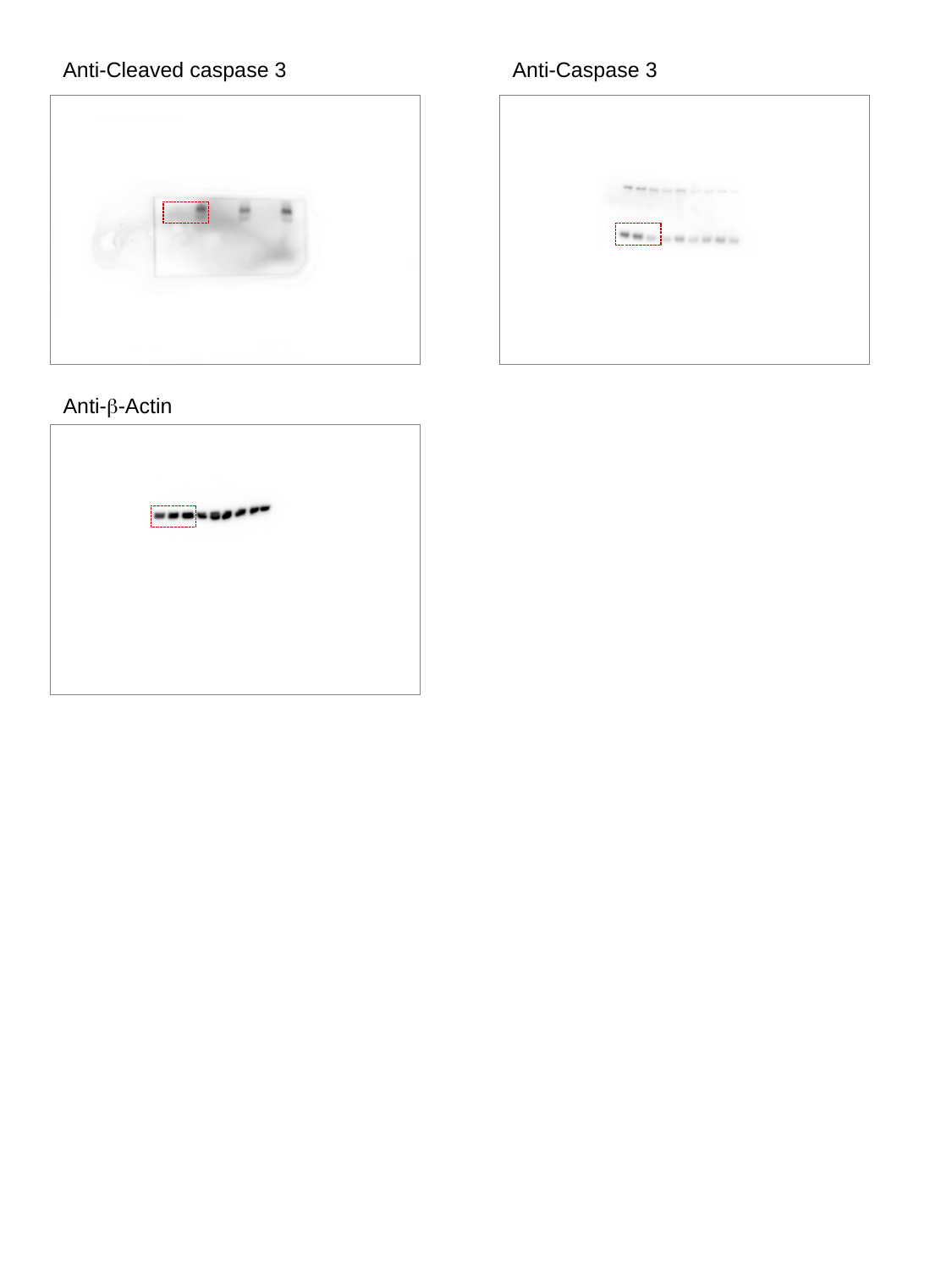

Anti-Cleaved caspase 3
Anti-Caspase 3
Anti-b-Actin

Supplement: Supplementary file 19 [file LSA-2025-03453_SdataFS8.3.pptx]

## Slide 1
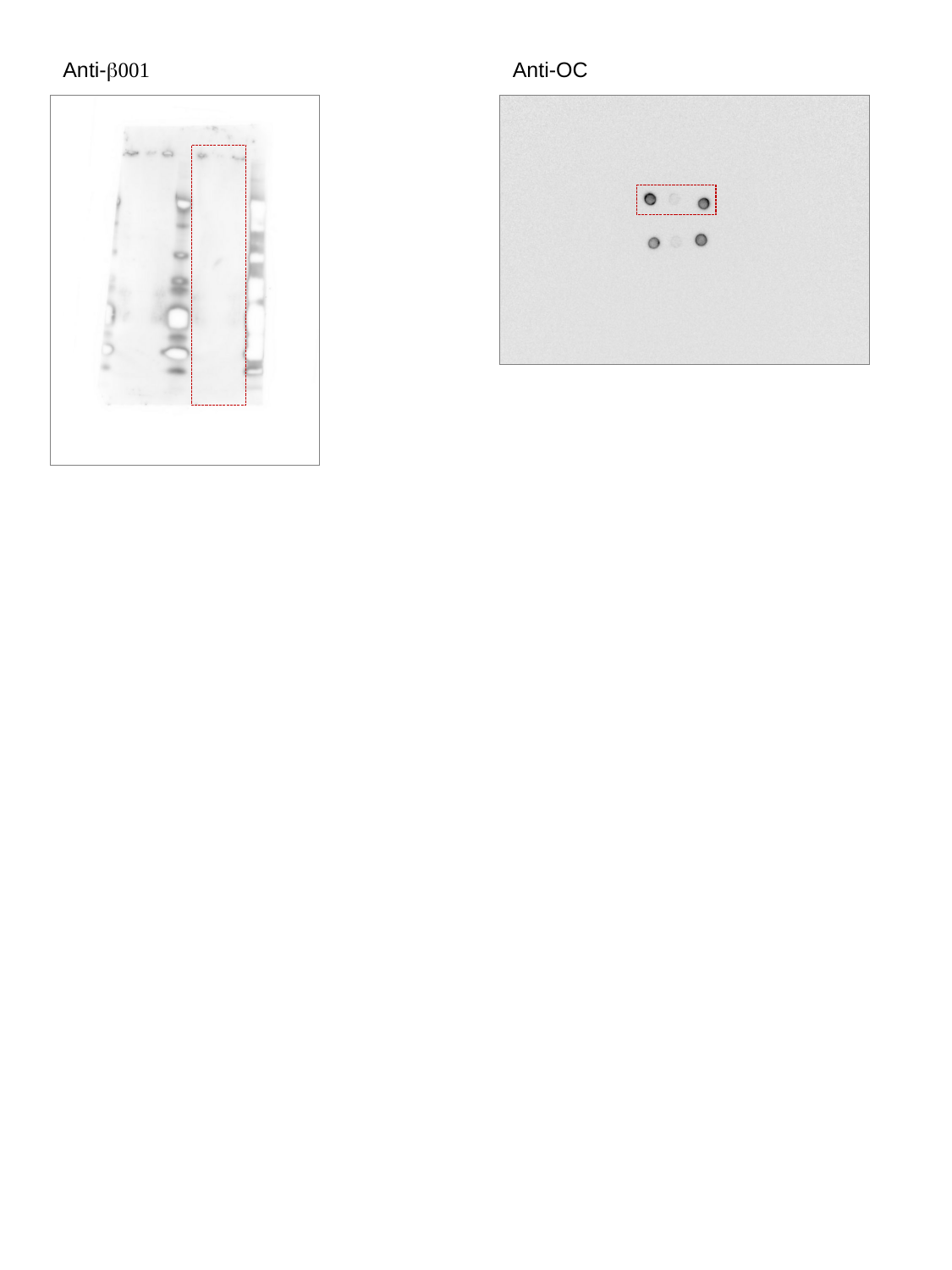

Anti-b001
Anti-OC

Supplement: Supplementary file 23 [file LSA-2025-03453_SdataFS10.2.pptx]
