# Supplementary material for: Amyloid-β fibrils accumulated in preeclamptic placentas suppress cytotrophoblast syncytialization
Source: Life Sci Alliance. 2026 Jan 20;9(4):e202503453. doi: 10.26508/lsa.202503453 (PMC12819053; doi:10.26508/lsa.202503453)
Supplement: Supplementary file 13 [file LSA-2025-03453_SdataF4.2.pptx]

## Slide 1
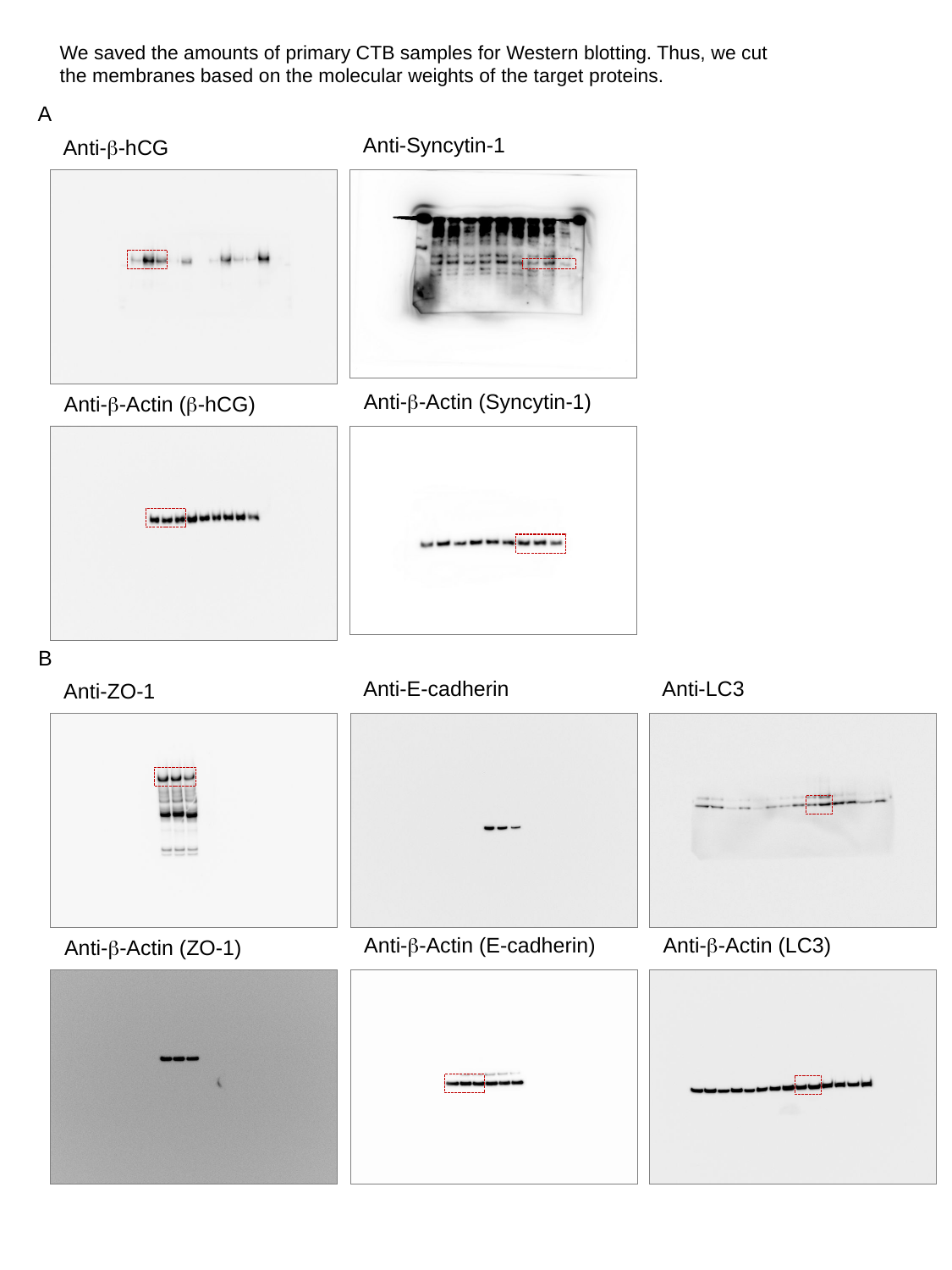

We saved the amounts of primary CTB samples for Western blotting. Thus, we cut the membranes based on the molecular weights of the target proteins.
A
Anti-Syncytin-1
Anti-b-hCG
Anti-b-Actin (Syncytin-1)
Anti-b-Actin (b-hCG)
B
Anti-E-cadherin
Anti-LC3
Anti-ZO-1
Anti-b-Actin (E-cadherin)
Anti-b-Actin (LC3)
Anti-b-Actin (ZO-1)
